# The International Hahnemannian Association

**Published:** 1899-08

**Authors:** 


### THE INTERNATIONAL HAHNEMANNIAN ASSOCIATION.

The twentieth annual convention of the International Hahnemannian Association was held at Niagara Falls, June 27th, 1899.

The convention was called to order by the President, Walter M. James, with the following members present: Dr. Wm. P. Wesselhœft, Dr. James Henry Allen, Dr. H. C. Allen, Dr. John Storer, Dr. H. W. Pierson, Dr. Clarence Willard Butler, Dr. T. Dwight Stow, Dr. B. L. Baylies, Dr. John Campbell, Dr. E. H. Kendall, Dr. S. L. Guild Leggett, Dr. Thomas L. Dillingham, Dr. S. F. Shannon, and the following visitors: Dr. Smith, editor of the *Medical Visitor;* Dr. Arthur Fisher, Dr. E. H. Wilsey, Dr. H. A. Mumaw, Dr. G. E. Waring.

The Secretary, Dr. E. E. Case, was absent owing to the critical illness of his father. In his absence Dr. Kendall, of Detroit, was made Secretary pro tem.

The President then read his address, already published in the July number of THE HOMŒOPATHIC PHYSICIAN.

The Necrologist, Dr. Storer, then read his report on deaths of members of the Association during the past year.

Dr. Wm. P. Wesselhœft made some remarks upon the death of Dr. Winn, of Boston.

The delegates' reports being in order, Dr. Storer reported as delegate from Dunham College; Dr. H. C. Allen from Hering College. Dr. Pierson reported from Engleside Society; Dr. Mumaw from Northern Indiana and Southern Michigan Society; Dr. Butler from New Jersey State Society.

A committee on the President's address was appointed, consisting of Drs. Wm. P. Wesselhœft, Clarence W. Butler, and H. C. Allen.

When this committee reported, discussion upon the points of the address was carried on by Drs. Wesselhœft, Butler, Stow, H. W. Pierson, and H. C. Allen.

Dr. Baylies, Chairman of the Board of Censors, made a report. There were eleven candidates for membership.

The Treasurer, Dr. Franklin Powel, was absent, owing to the death five days previously of a most estimable son, just turned twenty-one years of age, just come into possession of a handsome fortune, just about to enter the ministry, and shortly to be married to a charming young lady.

In the absence of the Treasurer, Dr. Butler was elected Treasurer pro tem., and instantly set to work to collect annual dues.

The report of the Bureau of Materia Medica being in order, the Chairman, Dr. Boger, being absent, Dr. H. C. Allen was appointed Acting Chairman.

Contributions to this Bureau were papers on Rhus-tox. and Rhus-radicans, by H. C. Allen; China, by C. M. Boger; "Scattered Gems," by C. M. Boger; Xantoxylum, by Dr. Boger; Ruta-Graveolens, by Dr. Fincke; X-Ray, by Dr. Fincke; "Confirmed Symptoms," by E. E. Case.

The Bureau of Homœopathic Philosophy was opened, and there were several contributions to this Bureau. Among them were the following: "Use and Relative Value of Symptoms," by S. L. Guild Leggett; "Actual and Potential Existence and Action," by J. Henry Allen.

Dr. Case's report as Secretary having been received, was read.

Dr. Lawrence Stanton, Corresponding Secretary, sent a report, which was read.

An amendment to the by-laws by which the words "Homœopathic treatment only of obstetrics and surgery" was inserted, was adopted.

The election of several members was confirmed.

Dr. H. C. Allen offered the thanks of the society to Dr.

Dillingham for his gift to the members of printed copies of the chart compiled by Dr. Boger.

The Bureau of Homœopathic Philosophy was opened, and in the absence of Dr. Yingling, Chairman of this Bureau, Dr. Wm. Wesselhœft was appointed Acting Chairman.

Dr. Stow read a paper on Clinical Cases, and followed it with an address upon the evils of vaccination.

Dr. Leggett read an admirable paper of Clinical Cases, which evoked very warm commendation.

Dr. Wesselhœft read a paper entitled "Chronic Cephalalgia with Chronic Catarrh."

A paper entitled "Malaria Officinalis," by Dr. W. A. Yingling, was read by Dr. J. Henry Allen.

Dr. Storer read a paper entitled "The Rational Feeding of Infants." He also read a paper on "Intermittent Fever with Mastoiditis."

Next on the programme was the report of the Bureau of Obstetrics. In the absence of the Chairman, Dr. Tomhagen, Dr. Stow was appointed Acting Chairman.

There were several papers contributed to this Bureau. Among them were :"Another Case of Painless Labor," by Dr. A. McNiel; "Gynæcology," by Dr. Majumdar, of Calcutta.

The Bureau of Surgery, Chairman, Dr. J. Henry Allen, was next opened. After which there was an election of officers, resulting in the choosing of the following:

President, Dr. James Henry Allen, of Chicago; Vice-President, Dr. S. L. Guild Leggett, of Syracuse, N. Y.; Secretary, Dr. E. E. Case, of Hartford, Conn.; Corresponding Secretary, Dr. Lawrence M. Stanton, of New York; Treasurer, Dr. Franklin Powel, of Chester, Pa.; Chairman of Board of Censors, Dr. B. L. B. Baylies, of Brooklyn, N. Y.; Necrologist, Dr. John Storer; Chairman of Bureau of Homœopathic Philosophy, Dr. B. Fincke, of Brooklyn, N. Y.; Chairman of Bureau of Materia Medica, Dr. E. H. Wilsey, of Parkersburg, W. Va.; Chairman of Bureau of Clinical Medicine, Dr. E. H. Kendall, of Detroit, Mich.; Chairman of Bureau of Obstetrics,

Dr. Clarence Willard Butler, of Montclair, N. J.; Chairman of Bureau of Surgery, Dr. Thomas L. Dillingham, of New York.

In response to the invitation of Governor Pingree, of Michigan, the next meeting will take place at Detroit, Mich., in June, 1900.

